# Assessing a respiratory toxic infectious bronchitis virus (IBV) strain: isolation, identification, pathogenicity, and immunological failure insights

**DOI:** 10.1128/spectrum.03990-23

**Published:** 2024-06-21

**Authors:** Huixin Liu, Chenchen Wang, Yang He, Xiaofang Wei, Junze Cheng, Wenwen Yang, Kaichuang Shi, Hongbin Si

**Affiliations:** 1 College of Animal Science and Technology, Guangxi Key Laboratory of Animal Breeding, Disease Control and Prevention, Guangxi grass station, Nanning, China; 2 Poultry Disease Diagnosis Division, Guangxi Center for Animal Disease Control and Prevention, Nanning, China; Institute of Microbiology Chinese Academy of Sciences, Beijing, China

**Keywords:** infectious bronchitis virus, isolated and identified, biological characteristics, sequence analysis, non-targeted metabolomics

## Abstract

Infectious bronchitis virus (IBV) is caused by avian coronavirus and poses a global economic threat to the poultry industry. In 2023, a highly pathogenic IBV strain, IBV/CN/GD20230501, was isolated and identified from chickens vaccinated with IBV-M41 in Guangdong, China. This study comprehensively investigated the biological characteristics of the isolated IBV strain, including its genotype, whole genome sequence analysis of its S1 gene, pathogenicity, host immune response, and serum non-targeted metabolomics. Through the analysis of the S1 gene sequence, serum neutralization tests, and comparative genomics, it was proven that IBV/CN/GD20230501 belongs to the GI-I type of strain and is serotype II. One alanine residue in the S1 subunit of the isolated strain was mutated into serine, and some mutations were observed in the ORF1ab gene and the terminal region of the genome. Animal challenge experiments using the EID_50_ and TCID_50_ calculations showed that IBV/CN/GD20230501 possesses strong respiratory pathogenicity, with early and long-term shedding of viruses and rapid viral spread. Antibody detection indicated that chickens infected with IBV/CN/GD20230501 exhibited delayed expression of early innate immune genes, while those infected with M41 showed rapid gene induction and effective viral control. Metabolomics analysis demonstrated that this virus infection led to differential expression of 291 ions in chicken serum, mainly affecting the citric acid cycle (tricarboxylic acid cycle).

**IMPORTANCE:**

This study identified an infectious bronchitis virus (IBV) strain isolated from vaccinated chickens in an immunized population that had certain sequence differences compared to IBV-M41, resulting in significantly enhanced pathogenicity and host defense. This strain has the potential to replace M41 as a more suitable challenge model for drug research. The non-targeted metabolomics analysis highlighting the citric acid cycle provides a new avenue for studying this highly virulent strain.

## INTRODUCTION

Infectious bronchitis virus (IBV) is a highly contagious viral respiratory disease that primarily affects poultry, particularly their respiratory system (1). Chickens infected with IBV often display respiratory symptoms such as wheezing, coughing, and difficulty breathing. The virus quickly spreads through the bloodstream and infects other organs (2). The severity of the infection and its impact on chickens’ health and productivity depend on the specific strain of the virus and the immune status of the host. Severe cases can lead to high mortality rates, reduced production performance, and egg failure (3). These issues, including reduced quantity and quality, result in significant economic losses to the poultry industry worldwide. IBV, a coronavirus that causes infectious bronchitis in poultry, belongs to the order Nidoviridae, the family Coronaviridae, the subfamily Orthocoronavirinae, and the genus Gammacoronavirus (4). It was first discovered in the United States in the 1930s, and since then, more than 50 IBV serotypes have been recorded (5). However, the cross-protective effect between different serotypes is limited, meaning that chickens infected with one serotype are not necessarily fully immune to other serotypes of IBV. The IBV virions have a linear, positive-sense, single-stranded genomic RNA with a 27.6 kb genome that contains at least 10 open reading frames (ORFs). These ORFs encode four structural proteins (spike glycoprotein, envelope protein, membrane glycoprotein, and nucleocapsid protein) and several nonstructural proteins (1). The two polyproteins encoded at the 5′ end of the genome (1a and 1ab) contain proteins required for RNA replication (6). Similar to other coronaviruses, the genetic diversity of IBV is generated through recombination events and mutations that occur during viral genome replication, including substitutions, deletions, and insertions (7, 8). This high proneness to mutation and recombination results in the existence of many types of this virus. In 2016, a classification system based on the molecular sequence diversity of the full-length S1 subunit was proposed, dividing IBV strains into seven genotypes (G1 to GVII) (9). China, for example, has at least eight genotypes of IBV circulating, including GI-1, GI-7, GI-13, GI-19, GI-22, GI-28, GVI-1, and GVII-1 (10). Furthermore, local lineage IBV variants have also been identified in chicken flocks in China, particularly in chickens with respiratory and secretory health problems (11–13).

Given the current trend of IBV strain mutation and its economic impact, the most effective measures for prevention and control still rely on the development of vaccines and broad-spectrum anti-IBV drugs (14–18). However, due to the high variability of IBV and limitations in cross-protection between serotypes, current vaccines are unable to provide comprehensive protection (19–21). In some poultry farms, cases of illness and even death have been reported in vaccinated chicken flocks due to IBV infection (13, 22). Existing drugs, such as forsythia suspensa (23), hypericin (24), ivermectin (25), garlic extract (26), Sambucus nigra extracts (27), houttuynia cordata (28), and astragalus (29), have shown effectiveness against the M41 strain of IBV. This indicates that M41 is the commonly used disease model in current research on anti-IBV drugs. The M41 strain, used in China for producing inactivated vaccines with IBV antigens, exhibits stable biological characteristics and good immunogenicity. However, during the production and inspection process of the vaccine, there are instances where the potency test results for chicken infectious bronchitis are found to be lower than the required standards.

Drug research experiments using M41 have shown a shorter course of disease and milder symptoms. In this study, chickens that were seriously ill or had died were selected from vaccinated chicken flocks. An IBV strain highly similar to M41 was isolated and extensively investigated for its biological characteristics, complete genomic sequence, antigenicity, pathogenicity, host immune response in specific pathogen-free (SPF) chickens, and metabolic analysis. The primary objective of this research is to identify and validate a highly virulent model surrogate strain. This will help in comprehending the scientific rationale and practical implications of how a high-virulence model strain can support the advancement of vaccines and drugs. Furthermore, the aim is to screen for model strains that closely mimic the virulence of wild-type pathogens to enhance the process of vaccine screening and drug evaluation. This will ensure the elicitation of adequate immune responses and the precise prediction of drug effects. Ultimately, this study seeks to gain insights into the protective mechanisms and safety profile of vaccines and drugs, contributing to their development and efficacy.

## RESULTS

### Isolation and identification of the virus

**Fig 1:**
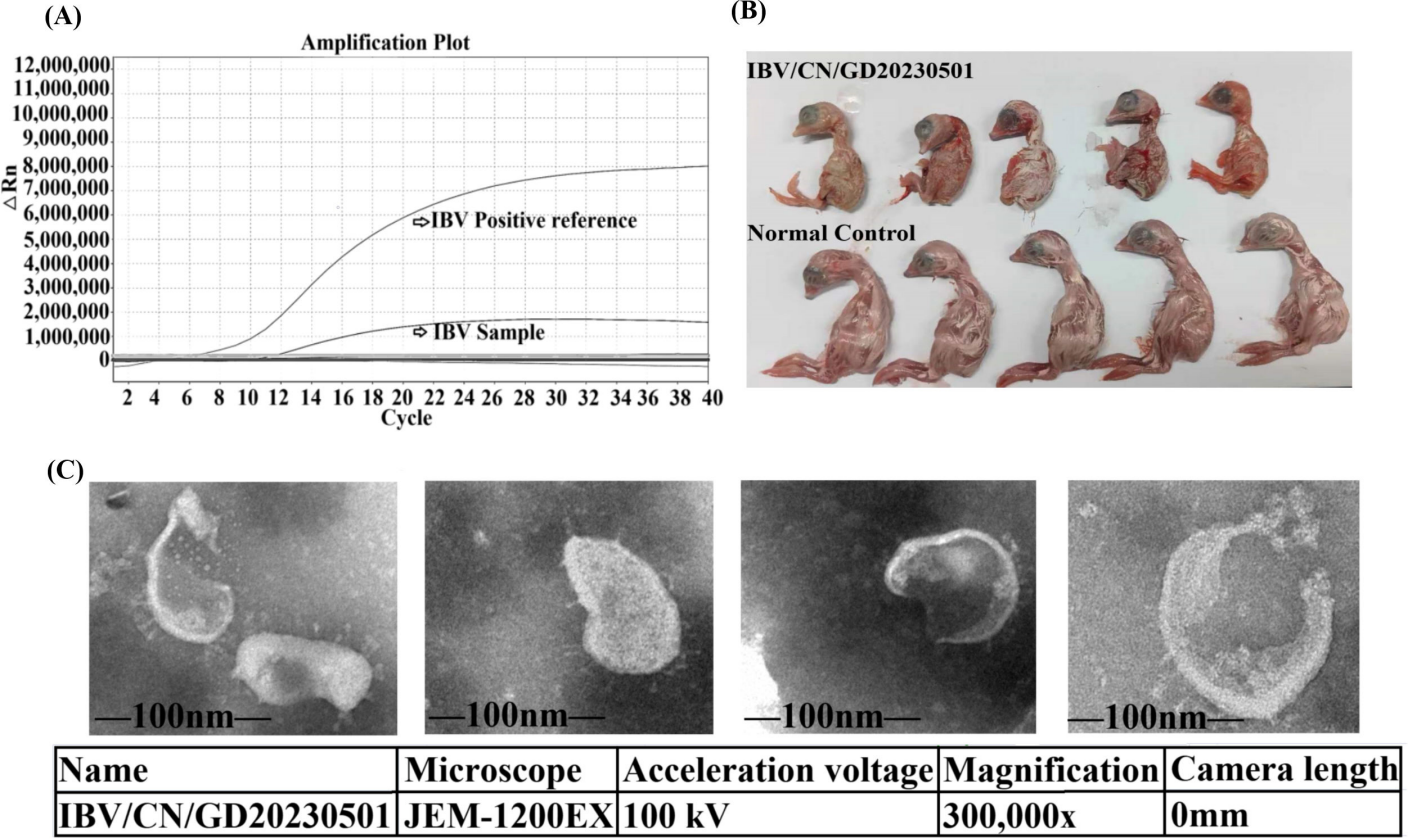
Isolation and identification of IBV/CN/GD20230501. (A) Fluorescent quantitative PCR amplification results of IBV in tissue. (**B**) Chicken embryo dwarfing test caused by IBV/CN/GD20230501 (72 hours). (**C**) Transmission electron microscopy detection of particles in allantoic fluid of IBV/CN/GD20230501.

The isolated virus strain was initially tested for agglutination with 1% chicken red blood cells using a conventional method, which did not show any agglutination. The presence of IBV was detected in the allantoic fluid using qPCR. The group used a previously constructed recombinant plasmid standard of IBV N protein as the positive control, while RNase-free water served as the negative control. The TaqMan fluorescent quantitative PCR amplification, conducted under optimal reaction conditions, exhibited a single fluorescent curve for IBV (Fig. 1A), confirming that the isolated virus was indeed IBV. Subsequently, a dwarfing test was performed on chicken embryos. The results revealed that chicken embryos started to die successively 48 hours after inoculation. Upon dissection, the dead embryos displayed dwarfism with enlarged kidneys, presenting a characteristic “dwarf embryo” phenomenon (Fig. 1B). Electron microscopy analysis revealed the presence of typical coronavirus particles, measuring 113.382–157.293 nm in diameter, with a capsule and a crown-like arrangement of rod-shaped protrusions on the surface, measuring 16.090–22.162 nm in diameter. These findings are consistent with the structural features of the virus observed in transmission electron microscopy (Fig. 1C).

### Genomic features of IBV/CN/GD20230501

**Fig 2:**
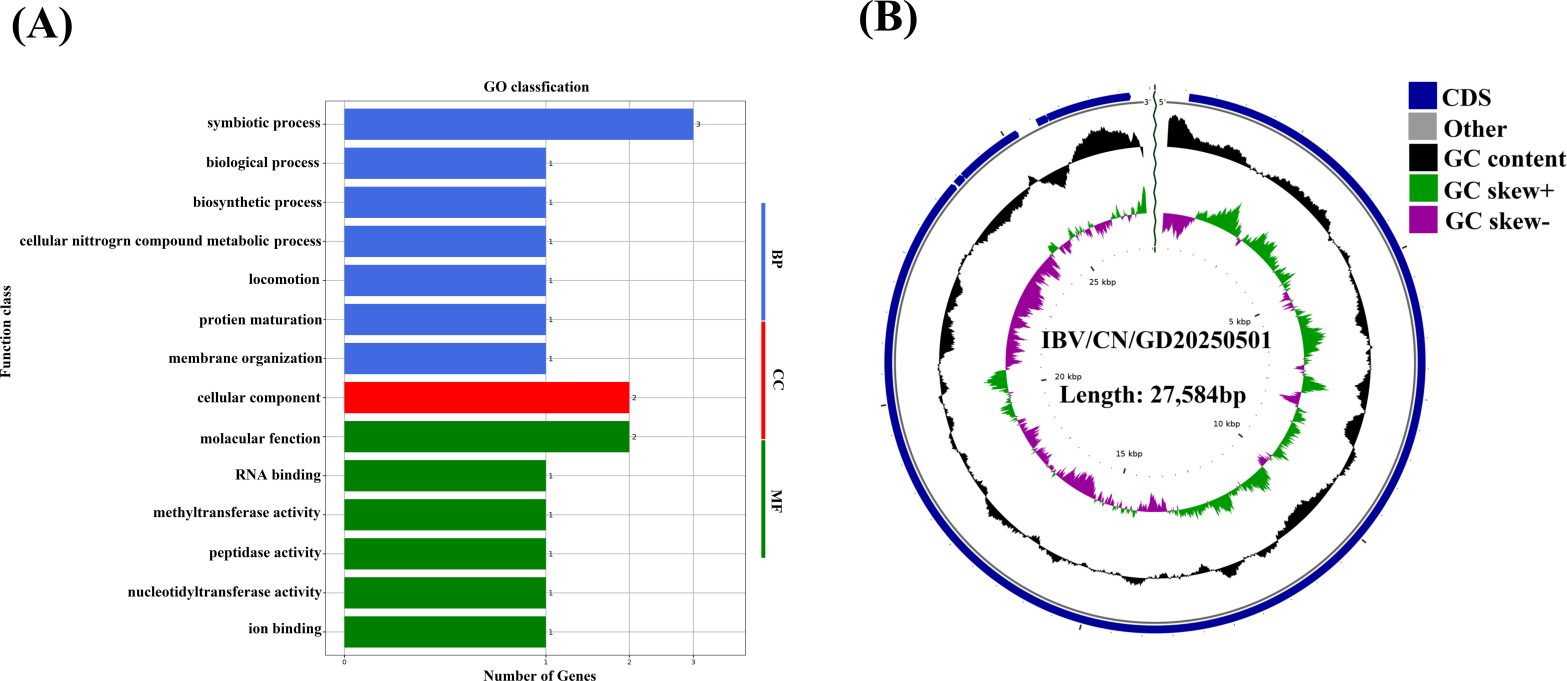
(**A**) The GO annotation of protein-coding genes, and (**B**) the IBV/CN/GD20230501 genome circle diagram. In the diagram, from the inside to the outside, the representation includes the scale, GC Skew, GC content, CDS/tRNA, and rRNA positions on the genome.

To understand the genomic features of the isolated strains, their entire genomes were fully sequenced, and the complete genomic sequences were submitted to GenBank under the accession number OR778292. The complete genome consists of 27,584 nucleotides, with a distribution of bases as follows: A, 28.94%; G, 21.8%; C, 15.94%; and T, 33.32%. This distribution is consistent with the classical IBV genomic structure of 5′-UTR-Pol-S-3a-3b-EM-5a-5b-N-UTR-3′. The ncRNA of IBV/CN/GD20230501 has four copy numbers, an average length of 52 bp, a total length of 208 bp, and accounts for 0.7541% of the genome. The total length of the open reading frame is 26,411 bp, with an ORF density of 0.326 genes per kb. The longest ORF length is 19,895 bp, and the average ORF length is 2,934.56 bp. The intergenic region length is 1,173 bp. The ORF/genome (coding percentage) is 95.75%, while the intergenic length/genome is 4.25%. The GC content in the ORF region is 37.75%, and the GC content in the intergenic region is 37.43%. The genes were annotated using InterPro software and GOSlim annotations in the Generic files (Fig. 2A). Based on this information, the gene circle map of the IBV/CN/GD20230501 genome was drawn (Fig. 2B).

Table 1 presents the gene division and protein-coding gene prediction for IBV/CN/GD20230501. To compare the protein-coding gene sequences with the protein sequences in the database, diamond blastp was used with a sequence comparison cut-off value of 1e-6. The protein function was determined based on the Hit name result. The analysis uncovered nine open reading frames within the virus, with the IBV M41 strain displaying the most significant hits. Protein function analysis of IBV/CN/GD20230501 was conducted by associating Swiss-Prot names with the respective protein-coding genes. ORF1ab is a multifunctional protein that includes a protease responsible for cleaving multiple proteins and participating in virus RNA transcription and replication. The spike protein is divided into S1 and S2 subunits. S1 attaches virus particles to the host cell membrane by interacting with sialic acid, triggering infection, while S2 acts as a class I virus fusion protein, mediating the fusion of virus particles and the cell membrane. The 3a protein contributes to resistance against interferon. The envelope protein plays a central role in virus morphogenesis and assembly. It acts as a viroporin, self-assembling in the host membrane to form a pentameric protein-lipid pore that allows for ion transport. Additionally, it induces cell apoptosis. The membrane protein is a component of the virus envelope and is involved in virus morphogenesis and assembly through interactions with other virus proteins. The 5b protein is responsible for host translation shutdown without degrading host RNA. By suppressing host gene expression, it helps the virus evade the host’s type I interferon immune response. The nucleocapsid protein packages the positive-strand virus genome RNA into a helical ribonucleoprotein. It plays a crucial role in virion assembly by interacting with the virus genome and membrane protein M, enhancing the transcription efficiency of subgenomic virus RNA and virus replication.

**TABLE 1:** Gene annotation of the complete genome sequence of IBV/CN/GD20230501 strain^*a*^

Gene		Genome position	Size (nucleotide)	Size (amino acid)	Hit name	Identity	E value
5′-UTR		1–563	563	/	/	/	/
ORF1ab	/	564–20458	19,895	6,631	P0C6Y3	99.83	0
	NSP2	564–2585	2,022	673	/	/	/
	NSP3	2586–7355	4,770	1,589	/	/	/
	NSP4	7356–8903	1,548	515	/	/	/
	NSP5	8904–9824	921	306	/	/	/
	NSP6	9825–10706	882	293	/	/	/
	NSP7	10707–10955	249	82	/	/	/
	NSP8	10956–11585	630	209	/	/	/
	NSP9	11586–11918	333	110	/	/	/
	NSP10	11919–12353	435	144	/	/	/
	NSP11-12	12354–15172	2,819	930	/	/	/
	NSP13	15173–16972	1,800	599	/	/	/
	NSP14	16973–18535	1,563	520	/	/	/
	NSP15	18536–19549	1,014	337	/	/	/
	NSP16	19649–20458	810	269	/	/	/
Spike protein		20409–23870	3,462	1,153	P12651	99.65	0
3a protein		23897–24070	174	57	P05137	98.25	1.57E-31
3b protein		24070–24264	195	64	P05138	100.00	6.31E-43
Envelope protein		24245–24574	330	109	P05139	98.17	5.20E-76
Membrane protein		24546–25223	678	225	P69607	100.00	1.56E-165
5a protein		25574–25771	198	65	Q5I5X4	98.46	1.88E-40
5b protein		25768–26016	249	82	Q80RZ3	100.00	7.11E-57
Nucleocapsid protein		25959–27188	1,230	409	Q98Y32	99.51	2.98E-292
3′-UTR		27189–27584	396	/	/	/	/

^*a*^ /, not applicable.

### Sequence analysis and complete genome

**Fig 3:**
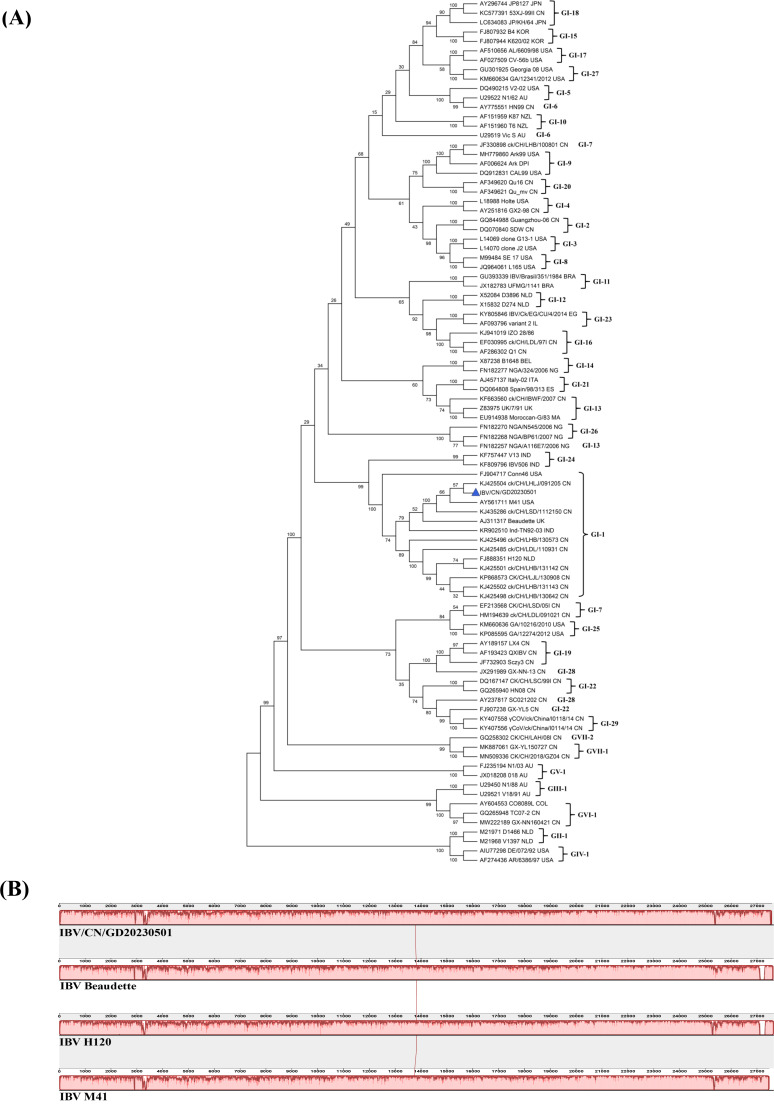
(**A**) The alignment of the IBV/CN/GD20230501S1 genome sequence. In panel **B**, collinearity analysis is presented, where blocks with similar colors indicate presumed homologous blocks without internal genome rearrangements. The top and bottom of the blocks represent the sense strand and antisense strand, respectively. The peak diagrams in different regions depict the similarity range of the sequence.

The phylogenetic tree based on the S1 gene is shown in Fig. 3. The results of the S1 phylogenetic tree indicate that the IBV/CN/GD20230501 isolate has the highest similarity with the Chinese isolate ck/CH/LHLJ/091205 and belongs to the GI-I type (Fig. 3A). Additionally, a base mutation (G to T) was observed at position 21,789 bp in the S1 sequence of IBV/CN/GD20230501, resulting in the replacement of alanine with serine. Comparing the genome characteristics of IBV/CN/GD20230501 with the classic strains IBV M41, IBV Beaudette, and IBV H120, it was found that there are significant differences in genome size, GC content, and protein coding. These differences mainly arise from individual base mutations, with no gene deletions or insertions in protein-coding genes (Table 2). Collinearity analysis of the genome sequences using Mauve software (version 2.3.1) revealed that IBV/CN/GD20230501 has the highest similarity with IBV M41, with only minor changes observed around 3,000 bp (NSP3), 15,000–16,000 bp (NSP13), and the terminal sequence (3′-UTR). No recombinant fragments were found in the strain (Fig. 3B). Serotype identification results indicate that both IBV/CN/GD20230501 and M41 strains have extremely high neutralizing titers, with a neutralizing titer of virus antiserum at 1:256, belonging to serotype II.

**TABLE 2:** Statistics of the genome characteristics

	IBV/CN/GD20230501	IBV M41	IBV Beaudette	IBV H120
Genome size (bp)	27,584	27,485	27,635	27,652
GC content (%)	37.7	37.8	37.9	38.1
Protein-coding genes	9	9	9	9
Protein coding (%)	95.16	95.50	95.06	94.80
Gene length (aa)				
1ab	6,631	6,631	6,629	6,611
S	1,153	1,153	1,162	1,162
3a	57	57	57	57
3b	64	64	64	64
3c	109	109	108	109
M	225	225	225	225
5a	65	65	65	65
5b	82	82	82	82
N	409	409	409	409

### Virus titer determination of IBV/CN/GD20230501

**Fig 4:**
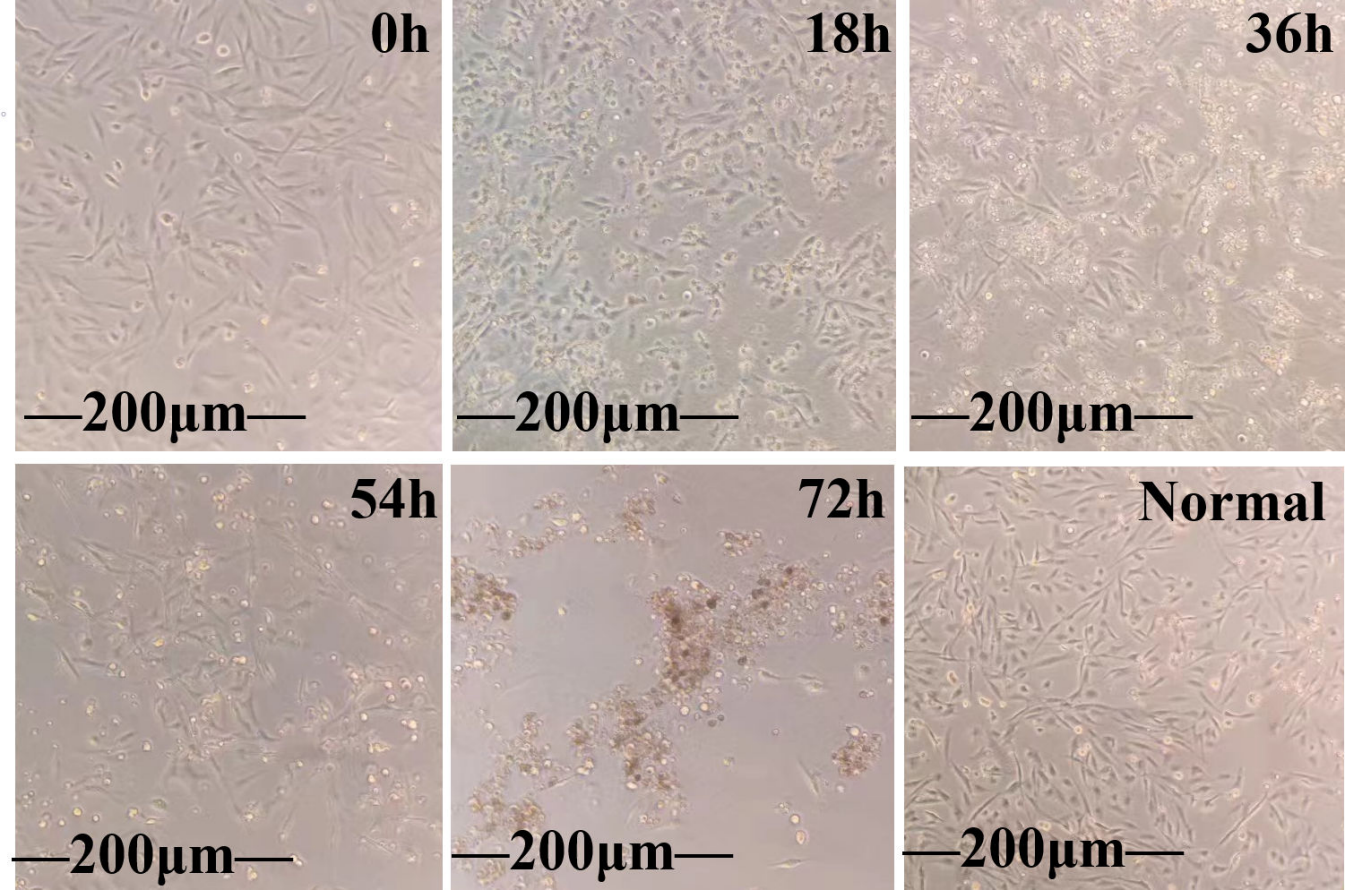
Observation of lesions after infection of CEK cells by the IBV/CN/GD20230501 strain.

CEK cells inoculated with IBV/CN/GD20230501 after 15 generations of blind transmission developed cytopathy effect (CPE). The virus collection was based on three repetitive freeze-thaw experiments conducted on ice, after which the samples were centrifuged at 12,000 rpm for 2 min to remove the supernatant and eliminate cellular debris, thus obtaining the viral supernatant. The 16th-generation virus supernatant was then inoculated into cell bottles filled with CEK cells. Changes in the cells were observed every 18 hours. Notable alterations in the cells were observed 36 hours post-infection, characterized by swelling and rounding of some cells, as well as a small number of cell ruptures. Over a period of 72 hours, the virus-infected cells gradually detached and accumulated (Fig. 4). The TCID_50_ of the IBV/CN/GD20230501 strain was determined (Table S1) and calculated to be 10^-5.67^/0.1 mL using the Reed-Muench method. SPF chicken embryos inoculated with the IBV/CN/GD20230501 strain showed no mortality within 24 hours. After being left undisturbed for 5 days, the embryos were dissected. Based on the pathological changes observed in the embryos, the EID_50_ was determined (Table S2) and calculated to be 10^-6.16^/0.1 mL using the Reed-Muench method.

### Enhanced pathogenicity of IBV/CN/GD20230501 in chickens

Sixty SPF chickens were randomly divided into three groups: the IBV/CN/GD20230501 group, the IBV-M41 group, and the blank control group, with 20 chickens in each group. To test the pathogenicity of the IBV/CN/GD20230501 strain, each 2-week-old SPF chicken was challenged by dripping 105 EID_50_ virus into the nasal cavity. Among the 20 chickens infected with IBV M41, 17 showed mild to moderate clinical symptoms such as cough and cold 4 days post-challenge, leading to a morbidity rate of 85%. All chickens infected with the IBV/CN/GD20230501 strain showed clinical symptoms, resulting in a morbidity rate of 100%. Additionally, 7 out of 20 chickens exhibited severe respiratory symptoms, distress, and depression 6 days after the challenge. No chickens died because of the disease during the experiment.

**Fig 5:**
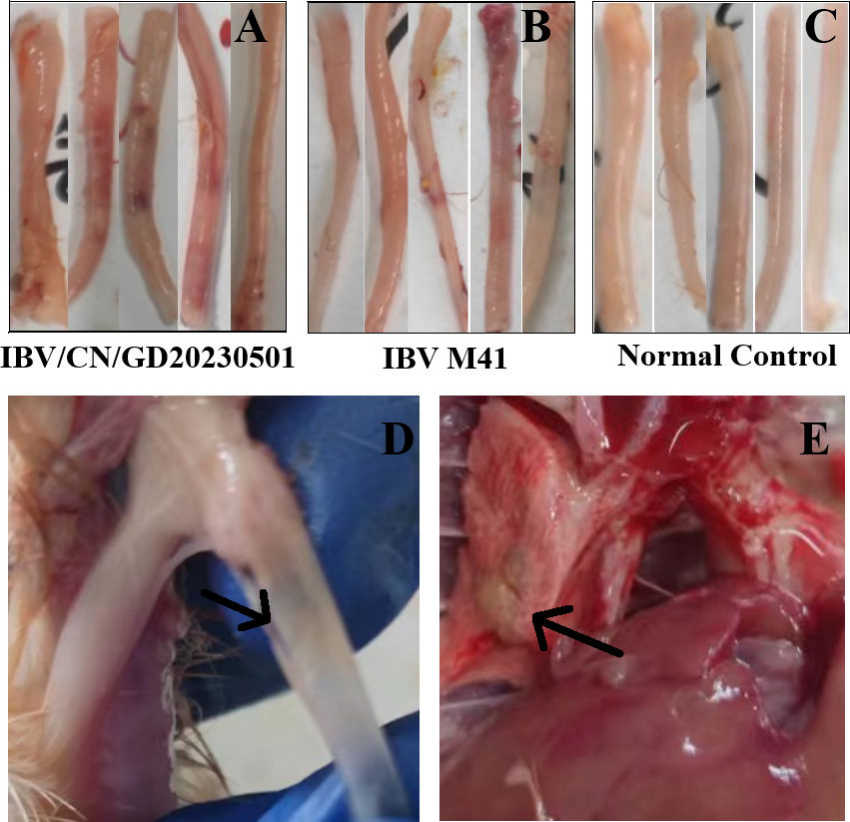
The pathological changes observed in chickens infected with IBV. Panels A–C depict chickens infected with IBV/CN/GD20230501 and show evident hemorrhagic tracheitis with blood clots. Panel D shows the presence of tracheal mucus in chickens infected with IBV. Panel E highlights the lung lesions observed in chickens infected with IBV/CN/GD20230501.

**Fig 6:**
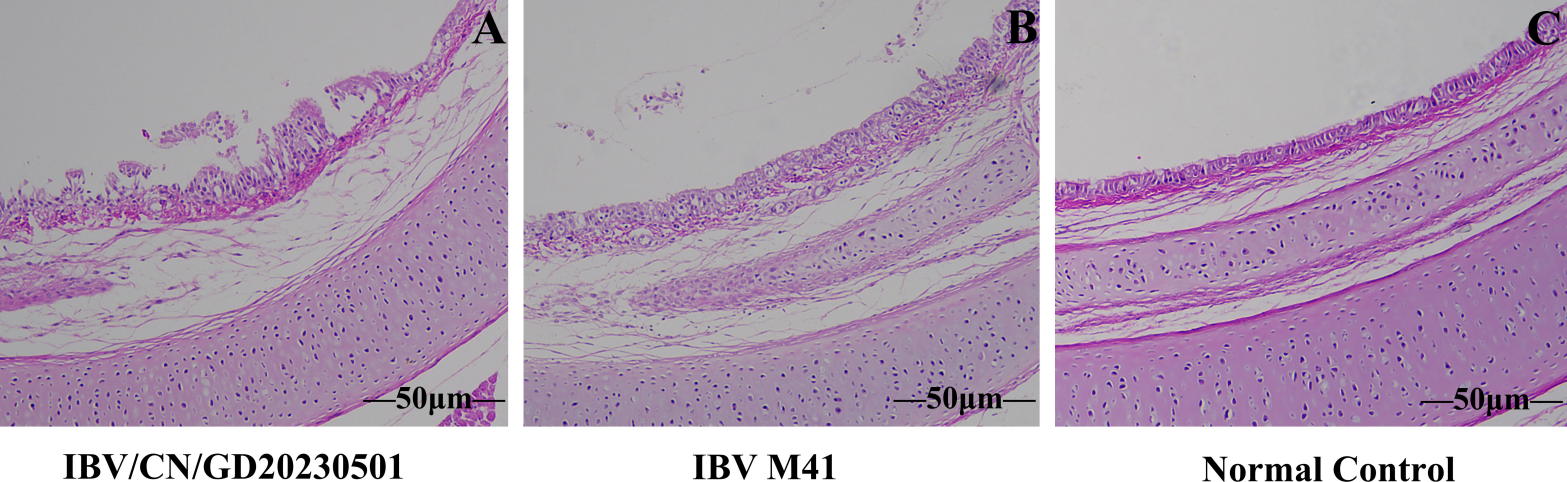
Histopathological lesions in the tracheal tissue of chickens infected with IBV. The tracheal tissue mucosa of groups A and B, corresponding to IBV/CN/GD20230501 and IBV M41, respectively, exhibited disordered characteristics such as detachment of ciliated cells, infiltration of heterophil cells, and proliferation of tracheal lymphocytes and epithelium. Specifically, the epithelial cells infected with IBV/CN/GD20230501 showed extensive shedding, degeneration, and necrosis, accompanied by a significant infiltration of lymphocytes.

To detect pathological changes after chickens were infected with the IBV/CN/GD20230501 strain, five chickens from each group were euthanized 7 days after the challenge, and their organs were dissected and observed. Compared with the phosphate-buffered saline (PBS) control group, three chickens infected with IBV-M41 and five chickens infected with the IBV/CN/GD20230501 strain showed obvious hemorrhagic tracheitis accompanied by blood clots (Fig. 5A through C). In addition, two chickens infected with IBV-M41 and three chickens infected with the IBV/CN/GD20230501 strain had evident mucus in the trachea (Fig. 5D). Furthermore, one chicken infected with the IBV/CN/GD20230501 strain showed significant pulmonary lesions (Fig. 5E). No pathological changes were observed in other organs, and there was no evident uric acid deposition in the kidneys. The control group did not exhibit any clinical signs or gross pathological changes. Histopathological examination revealed lymphocyte infiltration and epithelial cell shedding in the trachea after infection with the IBV/CN/GD20230501 strain and the IBV M41 strain, with a large number of lymphocytes infiltrating (Fig. 6). The trachea of the control group chickens did not show any changes.

### IBV/CN/GD20230501 spreads more rapidly and persistently in chickens

**Fig 7:**
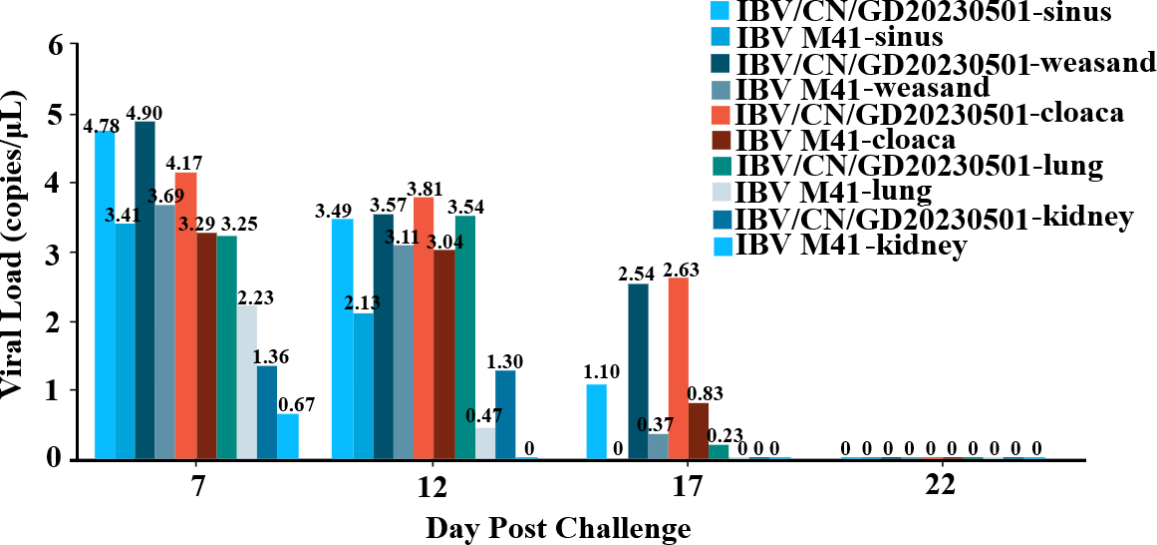
Viral organ load in various tissues of chickens infected with IBV, comparing the sinuses, trachea, cloaca, lungs, and kidney.

According to the previously constructed PCR detection method, we tested the viral load in various tissues of chickens at different time points (7, 12, 17, and 22 dpi). The results, shown in Fig. 7, revealed that the virus was detectable in the sinus and tracheal tissues of all infected chickens at 7 dpi. The viral genome copy numbers in the sinus and tracheal tissues of chickens infected with the IBV/CN/GD20230501 strain were 4.78 ± 0.75 and 4.90 ± 0.32 copies/μL, respectively. Furthermore, the virus content of the IBV/CN/GD20230501 strain was higher than that of IBV M41 at 7, 12, and 17 dpi, and the duration of virus excretion was longer. However, no virus could be detected in any group of chickens at 22 dpi.

### IBV/CN/GD20230501 infection causes lower antibody titers in chickens

**Fig 8:**
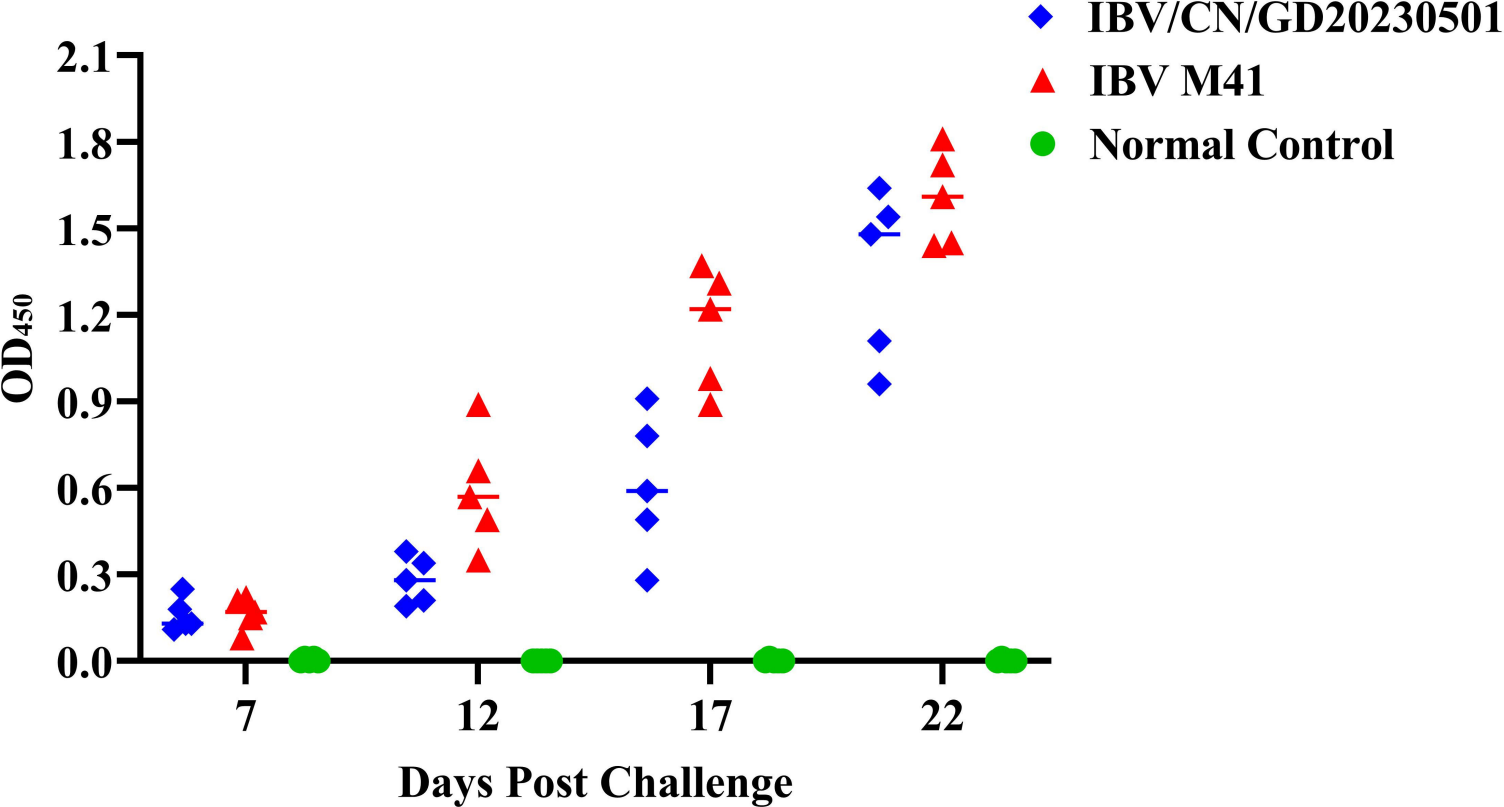
Detection of IBV-induced antibodies. To assess the immune response following infection with various types of IBV, serum samples were collected at 7-, 12-, 17-, and 22-days post-challenge. Antibodies were detected using ELISA, and samples with an OD_450_ value below 0.3 were classified as negative for anti-IBV antibodies.

To assess the immune response of chickens infected with different IBVs, we conducted IBV ELISA antibody tests on blood samples collected from the sub-wing veins of five chickens in each group at 7, 12, 17, and 22 dpi. Samples with an OD_450_ value below 0.3 were considered negative for anti-IBV antibodies (Fig. 8). The results revealed that all sera from chickens infected with IBV M41 tested positive for anti-IBV antibodies at 12 dpi, whereas all sera from the five chickens infected with the IBV/CN/GD20230501 strain tested positive within 21 days of infection. Compared to the classical vaccine strain IBV M41, the IBV/CN/GD20230501 strain of the same serotype exhibited higher virulence. The mortality rate was 0, and the production of antibodies occurred 5–10 days later than other strains, with a slower rise in antibody levels and lower antibody titers compared to the IBV M41 strain. Therefore, it can be considered a potential candidate strain for IBV respiratory attack modeling.

### Untargeted metabolomics of IBV/CN/GD20230501

**Fig 9:**
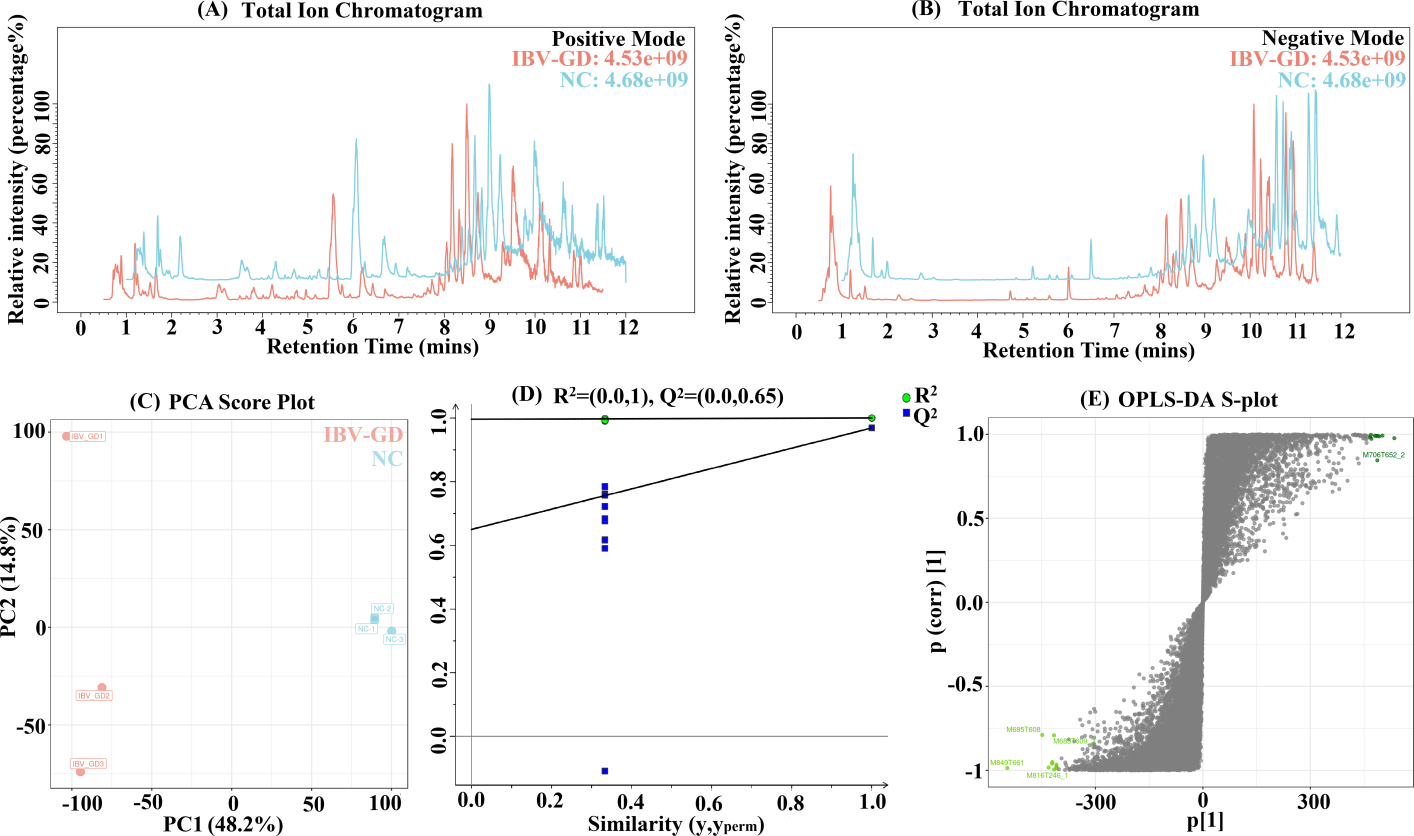
Data inspection of untargeted metabolomics. (A and B) Base peak chromatogram. (**C**) PCA score plot. (**D**) PLS-DA permutation test plot. (**E**) OPLS-DA S-plot.

Using LC/MS and untargeted metabolomics methods, we conducted an analysis of the metabolic mass spectrum of chicken serum to investigate potential indicators associated with changes induced by IBV/CN/GD20230501. The overlap of QC samples in both positive and negative ion modes demonstrates the stability of the system (Fig. 9A and B). The principal component analysis (PCA) reveals a stable and reliable model, as indicated by R2X(cum) = 0.61 > 0.5 (Fig. 9C). The goodness of fit (*R*2 = 0.619) and predictive ability (*Q*2 = 0.969) confirm that the sample is not overfitting (Fig. 9D). The S-plot highlights metabolites strongly correlated with the principal component in the orthogonal process (Fig. 9E). These findings collectively suggest that the model exhibits high reliability and predictability.

**Fig 10:**
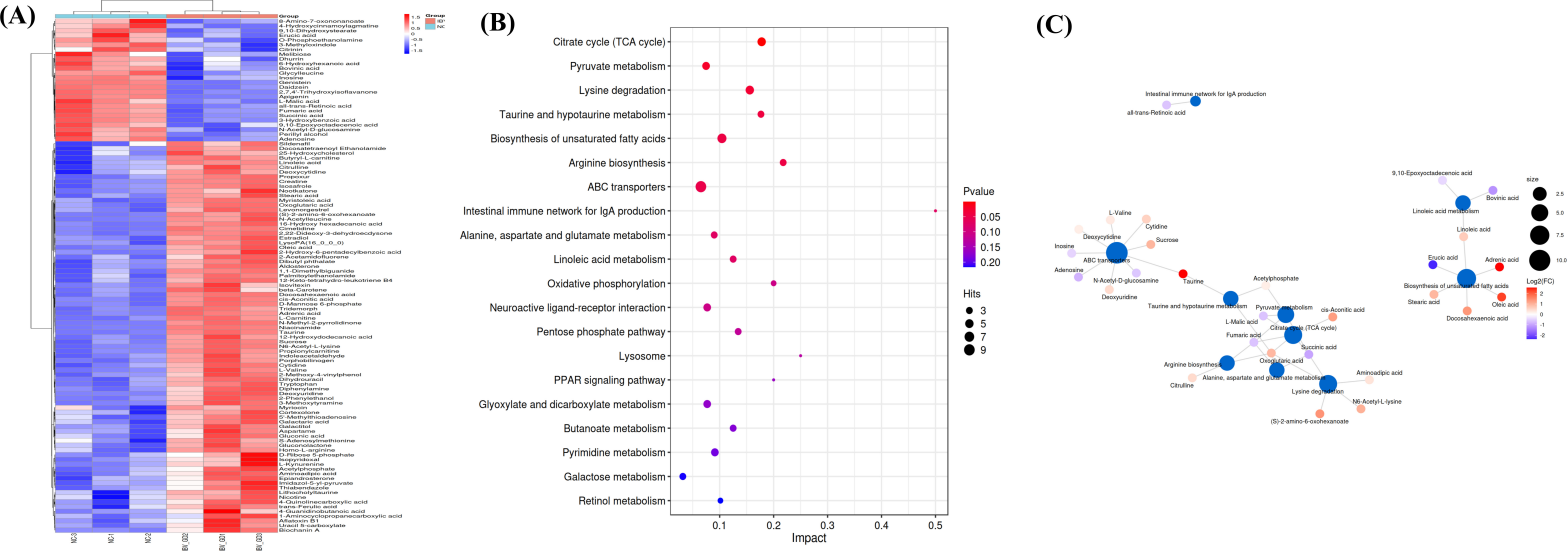
The results of the differential metabolite and KEGG pathway enrichment analysis. In panel A, a clustering heat map is displayed, with columns representing samples and rows representing metabolites. The clustering tree on the left indicates the differential metabolite clustering, while the top tree represents the sample clustering. The color gradient represents the quantitative value, with redder colors indicating higher expression levels and bluer colors indicating lower expression levels. If the number of metabolites exceeds 150, their names are not displayed. Panel B shows a bubble chart depicting the factors influencing metabolic pathways. The *x*-axis represents the impact value enriched in different metabolic pathways, while the *y*-axis represents the enriched pathways. The size of each point corresponds to the number of metabolites associated with the pathway, and the color reflects the *P* value. Redder colors indicate smaller *P* values, while bluer colors indicate larger *P* values. Finally, in panel C, a network diagram is presented. Blue dots represent pathways, while other dots represent metabolites. The size of a pathway point indicates the number of metabolites connected to it. The color of the metabolite point represents the log2(FC) value, with red indicating differential upregulation, blue indicating differential down-regulation, and darker colors indicating greater differences.

Differential metabolites were identified from the primary metabolite list using a set statistical test method. The screening criteria included a *P* value less than 0.05 and a variable importance for the projection (VIP) value greater than 1 (30). A total of 21,674 metabolites were analyzed, resulting in the identification of 5,817 upregulated differential metabolites and 1,831 downregulated differential metabolites. Additionally, 7,648 differential metabolites were identified overall (Table S3). Further analysis and matching with the metabolite database led to the identification of 291 metabolites, primarily belonging to categories such as carboxylic acids and derivatives, fatty acyls, organooxygen compounds, benzene and substituted derivatives, and steroids and steroid derivatives (Fig. 10A; Table S4). KEGG pathway enrichment analysis, performed using MetaboAnalyst (www.metaboanalyst.ca), revealed that the abnormal metabolites affected various metabolic pathways, with the citrate cycle [tricarboxylic acid (TCA) cycle] being particularly notable (31). The analysis aimed to assess the significance of the given genes or metabolites in biological reactions based on their position in the pathway (Fig. 10B and C; Table S5).

## DISCUSSION

Infectious bronchitis virus is known to cause respiratory diseases in chickens, leading to significant economic losses in the poultry industry. The virus poses a challenge to the industry due to its rapid spread and multiple serotypes, which results in poor cross-protection (32–34). Previous reports have suggested that the weak protection offered by commercial Mass-type vaccines contributes to the spread of IBV (35). Therefore, it is essential to isolate and identify the IBV in order to gain insights into its epidemiology and pathogenic characteristics. By conducting isolation and identification studies, we can better understand the genome structure, serotype, and genetic variation of different IBV strains. This provides insight into the genetic diversity, evolutionary mechanisms, and antigenic variation of IBV. The strain examined in this study was obtained from immunized chicken farms (20). It was first isolated and identified in chicken embryos, displaying typical dwarf curling. Subsequent laboratory screenings for other avian diseases confirmed that the isolated strain was a single IBV, as observed through a transmission electron microscope, revealing a virus particle with a coronal garland formation after limit dilution.

Through high-throughput whole-genome sequencing, it was discovered that the IBV/CN/GD20230501 strain has a complete genome consisting of 27,584 nucleotides. It shares a similarity of 99.96% with the M41 vaccine strain and has not undergone recombination. Among the four structural protein genes of IBV, the S1 gene is highly prone to mutation, which is closely associated with serotype, pathogenicity, and the production of neutralizing antibodies (36). Therefore, genetic analysis of IBV primarily focuses on the S1 gene (9, 37). In this study, the complete S1 gene was utilized for IBV typing, revealing that the IBV/CN/GD20230501 isolate strain has the highest similarity to the Chinese isolate strain ck/CH/LHLJ/091205, belonging to the GI-I type. Notably, this genotype corresponds to the vaccine used in the chicken farm, thus supporting reports suggesting that newly identified highly homologous strains may originate from the Mass vaccine used in chicken farms (20). Upon comparing the genomes of the IBV/CN/GD20230501 isolate strain and M41, certain differences were observed, such as a mutation from alanine to serine in the S1 gene and some mutations at the end of the ORF1ab gene and the genome. Consequently, it cannot be ruled out that these changes contribute to immunization failure. Generally, strains of the same genotype are more likely to belong to the same serotype (38). Therefore, a cross-neutralization reaction experiment was conducted using M41-positive serum, which confirmed that both IBV/CN/GD20230501 and M41 strains exhibit extremely high neutralization titers, with a virus neutralization titer of 1:256, indicating serotype II. Although the amino acid mutation in the S1 gene did not result in a new genotype, it remains unclear whether it has enhanced immune escape (10). Moreover, previous studies have demonstrated that even a few amino acids or point mutations can impact biological activity (22). The IBV/CN/GD20230501 strain exhibits a mutation from alanine to serine in S1, along with mutations in the genome ORF1ab and the end of the gene. Previous reports from the UK have also identified similar findings, suggesting the presence of three different serotypes of IBV strains. The amino acid variation in the 19–122 and 251–347 regions of the S1 subunit is only 2%, which results in the failure of cross-protection (39). Studies on murine hepatitis virus, a group II coronavirus, have demonstrated that even single amino acid substitutions in the S protein can confer resistance to neutralization by S1 subunit-specific monoclonal antibodies (40). The spike protein is known to play a crucial role in determining the cellular tropism and pathogenicity of viruses (13, 41, 42). While current research primarily focuses on genes, mutations in non-coding regions can also have a significant impact. For instance, changes in the promoter region can lead to alterations in transcription (43). Therefore, it is essential to consider genetic changes beyond structural protein genes when evaluating the effectiveness of antigens from a single epitope, such as the spike protein, in inducing vaccine protection (13). The high degree of homology between the spike protein genes of the IBV/CN/GD20230501 strain and IBV M41 highlights the notable finding of increased virulence in the IBV/CN/GD20230501 strain.

A comparative analysis of its pathogenicity was performed to characterize the new strain IBV/CN/GD20230501. The IBV chicken embryo-adapted viruses, as well as the virus passaged multiple times through chicken embryos, were examined for their ability to form plaques and induce cytopathic effects in chicken kidney cells, chicken embryo kidney cells, and chicken embryo cells (44). The isolated strain was initially passaged through chicken embryos to enhance its adaptability to CEK cells. Cytopathic effect (CPE) were observed after 15 generations of blind passage in CEK cells. The 15th-generation virus fluid was added to the CEK cells, and its TCID50 was determined. The result was a TCID_50_ of 10^-5.67^/0.1 mL, which is approximately equivalent to an EID50 of 10^-6.16^/0.1 mL.

In animal pathogenicity trials, both IBV/CN/GD20230501 and M41 exhibited similar clinical symptoms, pathological dissections, tissue lesions, and mortality rates when tested on 14-day-old SPF chickens at the same dosage. However, chickens infected with IBV/CN/GD20230501 showed stronger respiratory symptoms compared to those infected with M41. The incidence rate of respiratory symptoms was 100% for IBV/CN/GD20230501, while the mortality rate was 0%. In field cases, the incidence rate was 90% with a mortality rate of 3%. This difference could be attributed to the high density of chicken farming and severe environmental pollution, which create conditions favorable for secondary infections by *Staphylococcus aureus* and *Escherichia coli* through respiratory infections, castration wounds, and horizontal transmission (22). Virus load in various organs was measured at different time intervals, revealing that IBV/CN/GD20230501 had higher virus titer in all organs compared to M41. The trachea had the highest virus content for both strains, and the virus was expelled through the nasal cavity via the respiratory tract. The quantity of virus expelled through the nasal cavity decreased as the disease progressed. Although high titers of the virus were found in cloacal swabs, no clinical diarrhea was observed, indicating another potential source of persistent infection. The strong virulence, respiratory symptoms, and prolonged virus shedding of the IBV/CN/GD20230501 strain require attention.

The IBV/CN/GD20230501 strain, although highly similar to the M41 strain, demonstrates greater virulence and a longer disease course. Additionally, it exhibits delayed antibody generation and seroconversion times compared to other strains, with a slower rise in antibody levels and lower antibody titers. Currently, the M41 strain is commonly used in antiviral experiments; however, its shorter disease course limits the effectiveness of safety testing for drugs. Therefore, the IBV/CN/GD20230501 strain could serve as an excellent model for investigating the molecular determinants of replication and pathogenicity of infectious bronchitis virus in chickens. Furthermore, it has the potential to replace the M41 strain in antiviral drug research.

Untargeted metabolomics revealed that the main metabolic pathway caused by the IBV/CN/GD20230501 strain in the host is the TCA cycle. The TCA cycle is a key part of energy production in host cells, and its disruption could significantly affect the energy balance of the host. We hypothesize that IBV infection may lead to an energy supply shortage in host cells by altering the TCA cycle, which might be reflected in the host cells’ efforts to maintain energy balance by adjusting other metabolic pathways. This metabolic adaptation could have profound effects on host cell function and the viral life cycle. The metabolic disturbances revealed in our study, particularly those affecting the TCA cycle, may offer new therapeutic strategies. Antiviral strategies could consider targeting those key metabolic nodes that the virus exploits to support its lifecycle. For example, if a specific enzyme in the TCA cycle is significantly affected by IBV infection, inhibiting the activity of this enzyme with drugs may reduce the availability of metabolites needed for viral replication, thereby inhibiting viral replication.

This study aimed to isolate an IBV strain from a chicken farm where the disease occurred after immunization. Whole-genome sequencing was performed to compare the isolated strain with the vaccine strain. Despite the high similarity between these strains, significant differences in pathogenicity in chickens were observed for the infectious bronchitis virus. These differences may be attributed to a proline-to-serine mutation in S1, as well as some mutations in the ORF1ab gene and at the end of the gene. The study hypothesizes that these mutation sites in the proteins affect the growth characteristics, virulence, and pathogenicity of the infectious bronchitis virus by influencing protein function. Future studies will focus on identifying the specific amino acids associated with virulence and pathogenicity in these proteins. Moreover, this research serves as a foundation for selecting matching IBV epidemic strains for modeling, thereby providing a scientific basis for effective control and prevention of infectious bronchitis. Ultimately, this contributes to the overall health and development of poultry farming.

## MATERIALS AND METHODS

### Cells and experimental animals

CEK cells (GeneO, Guangzhou, China) were cultured in Dulbecco’s Modified Eagle Medium (Solebao, China) supplemented with 10% fetal bovine serum (Hyclone, sourced from Australia), penicillin (250 U/mL), and streptomycin (250 µg/mL). The cells were incubated at 37°C in a humidified incubator with 5% carbon dioxide. SPF white-feathered broiler eggs at the age of 7 days were obtained from Guangxi Veterinary Research Institute, Guangxi Province, China. The eggs were incubated at 37.5°C with a humidity of 65%. Tracheal ring verification in the experiment was performed using SPF chicken embryos purchased and cultured until they reached 20 days of age. These embryos were placed in a culture medium containing 10% fetal bovine serum (Hyclone, sourced from Australia), penicillin (250 U/mL), and streptomycin (250 µg/mL) and cultured at 37°C in a humidified incubator with 5% carbon dioxide. One-day-old yellow-feathered broilers were obtained from Fufeng Company, Guangxi Province, China.

### Virus isolation and purification

The virus was isolated in March 2023 from local chicken breeds in Guangzhou, China. These chickens exhibited significant symptoms of acute respiratory disease at 30 days of age. The morbidity rate among the diseased birds was 90%, with a mortality rate of 3%. A gross examination of the affected birds revealed the presence of serous exudates in the trachea. To preliminarily identify the single infection with IBV, various tests, including the blood clot test and qPCR detection, were conducted. Additionally, other pathogens were excluded from the collected diseased materials. Trachea, lung, and kidney tissues were collected from the diseased birds under sterile conditions. These tissues were then ground, filtered through a bacterial membrane, and inoculated into 10-day-old SPF chicken embryos via the allantoic cavity route for three blind passages. Monoclonal IBV was obtained through limiting dilution passaging, following previous protocols (45). This involved inoculating 0.1 mL of allantoic fluid, diluted to various ratios ranging from 10^1^ to 10^9^, into 10-day-old SPF chicken embryos (five embryos per dilution). The collected allantoic fluid was then tested for IBV using RT-PCR. The allantoic fluid that tested positive for IBV and had the highest dilution ratio was used for the next dilution passage. After three rounds of limiting dilution passaging, the allantoic fluid that tested positive for IBV was selected for further analysis, including serotype identification, whole-genome sequencing, and pathogenicity testing.

### Biological characterization of isolated strain

The serotype of the isolate was determined using the tracheal organ culture (TOC) virus neutralization (VN) test (46). For this purpose, IBV M41-positive serum (Serotype II, Chasing Biology, China) was obtained and used in the preparation of chicken embryo TOCs. The median infectious dose in tracheal organ culture (TOC-ID50) was determined, along with the VN detection. The highest serum dilution of the neutralizing virus and the corresponding neutralizing titer causing ciliary congestion and ciliary beating were observed.

Embryo development obstruction experiments were conducted to investigate the impact of isolated strains. The strains were inoculated into 10-day-old SPF embryos through the allantoic cavity, with an inoculation volume of 0.2 mL/embryo. Each isolated strain was inoculated into 10 embryos. A control group was also established, where 10 embryos were inoculated with 0.85% physiological saline in the same volume. The embryos were then placed in a chicken embryo incubator, and their development was observed after 7 days of incubation.

To confirm the virus morphology, the IBV-positive allantoic fluid was first precipitated with a saturated ammonium sulfate solution. The resulting precipitates were then collected on a 30%–60% sucrose density gradient medium using ultracentrifugation. Next, the precipitates on each gradient interface were diluted with an appropriate amount of PBS and centrifuged at 70,000 r/min for 1 hour. After centrifugation, the precipitates were resuspended in TNE buffer and negatively stained with phosphotungstic acid. Finally, the samples were observed under a transmission electron microscope.

### Whole-genome sequencing and analysis

RNA was extracted from 200 µL of IBV isolate allantoic fluid using a StarSpin Fast Virus DNA/RNA Kit (Genstar, P144-01). Reverse transcription was performed using a StarScript III All-in-one RT Mix with gDNA Remover (Genstar, A230-10). The sequencing of the viral genome was carried out using the whole genome shotgun strategy on the Illumina NovaSeq platform by Pacino Bio-Technology Co., Ltd. (Guangzhou, China). The assembled sequences were analyzed using A5-MiSeq (47) and SPAdes (48), and collinearity analysis was conducted using MUMmer (49). The obtained results were corrected using Pilon to obtain the final viral genome sequence (50). Functional component analysis of the entire genome of the isolate was performed. Non-coding RNA prediction was mainly accomplished through comparison with the Rfam database (51). Protein-coding genes in the viral genome were predicted using GeneMarkS software (52), and GO annotation was completed using InterPro software (53). GOSlim annotation was performed using map2slim in the Generic file. The genome sequence, gene prediction, and prediction of non-coding RNA were integrated, and a circular map of the genome was generated using cgview (54). To determine the strain genotype, the sequence of the S1 gene was compared with 91 reference strains retrieved from the GenBank database (Table 3). A phylogenetic tree of the S1 gene was constructed using MEGA X software with the neighbor-joining method and 1,000 bootstrap replicates. The amino acid changes in the S1 gene were also analyzed using the same software. Genomic feature statistics and collinearity analysis were performed by comparing the full sequence of IBV/CN/GD20230501 with the classic strains of the same genotype listed in Table 3 (http://darlinglab.org/mauve/mauve.html).

**TABLE 3:** IBV genotypes and clustering reference strains^*a*^

Strain	Country	Genotype	Accession number	Location
H120	NLD	GI-1	FJ888351	20314–23802
M41	USA	GI-1	AY561711	1–1610 (S1)
Conn46	USA	GI-1	FJ904717	20371–23850
Ind-TN92-03	IND	GI-1	KR902510	20374–23835
ck/CH/LHLJ/091205	CN	GI-1	KJ425504	20374–23862
ck/CH/LSD/1112150	CN	GI-1	KJ435286	20374–23862
Beaudette	UK	GI-1	AJ311317	20368–23856
ck/CH/LDL/110931	CN	GI-1	KJ425485	20314–23802
ck/CH/LHB/130573	CN	GI-1	KJ425496	20314–23802
CK/CH/LJL/130908	CN	GI-1	KP868573	20302–23790
ck/CH/LHB/131143	CN	GI-1	KJ425502	20314–23802
ck/CH/LHB/131142	CN	GI-1	KJ425501	20314–23802
ck/CH/LHB/130642	CN	GI-1	KJ425498	20314–23802
Guangzhou-06	CN	GI-2	GQ844988	1–1611 (S1）
SDW	CN	GI-2	DQ070840	1–1623 (S1）
Clone G13-1	USA	GI-3	L14069	1–1738 (S1）
Clone J2	USA	GI-3	L14070	1–1738 (S1）
Holte	USA	GI-4	L18988	1–1636 (S1）
GX2-98	CN	GI-4	AY251816	1–1633 (S1）
N1/62	AU	GI-5	U29522	1–1709 (S1)
V2-02	USA	GI-5	DQ490215	1–3510
HN99	CN	GI-6	AY775551	1–1739 (S1）
Vic S	AU	GI-6	U29519	1–1703 (S1）
CK/CH/LSD/05I	CN	GI-7	EF213568	1–1614 (S1)
ck/CH/LDL/091021	CN	GI-7	HM194639	1–1807 (S1)
ck/CH/LHB/100801	CN	GI-7	JF330898	20420–23911
SE 17	USA	GI-8	M99484	1–1853 (S1)
L165	USA	GI-8	JQ964061	1–1632 (S1)
Ark99	USA	GI-9	MH779860	20332–23838
Ark DPI	USA	GI-9	AF006624	1–1632 (S1)
CAL99	USA	GI-9	DQ912831	1–1632 (S1)
K87	NZL	GI-10	AF151959	1–1635 (S1)
T6	NZL	GI-10	AF151960	1–1635 (S1)
IBV/Brasil/351/1984	BRA	GI-11	GU393339	1–1650 (S1)
UFMG/1141	BRA	GI-11	JX182783	1–1613 (S1)
D3896	NLD	GI-12	X52084	1–1776 (S1)
D274	NLD	GI-12	X15832	67–1680 (S1)
NGA/A116E7/2006	NG	GI-13	FN182257	1–1614 (S1)
ck/CH/IBWF/2007	CN	GI-13	KF663560	20361–23837
UK/7/91	UK	GI-13	Z83975	1–1617 (S1)
Moroccan-G/83	MA	GI-13	EU914938	1–1764 (S1)
B1648	BEL	GI-14	X87238	1–3503
NGA/324/2006	NG	GI-14	FN182277	1–1614 (S1)
B4	KOR	GI-15	FJ807932	1–1632 (S1)
K620/02	KOR	GI-15	FJ807944	1–1632 (S1)
CK/CH/LDL/97I	CN	GI-16	EF030995	1–1666 (S1)
Q1	CN	GI-16	AF286302	1–1626 (S1)
IZO 28/86	ITA	GI-16	KJ941019	1–1780 (S1)
AL/6609/98	USA	GI-17	AF510656	1–1647 (S1)
CV-56b	USA	GI-17	AF027509	1–1626 (S1)
JP/KH/64	JPN	GI-18	LC634083	20372–23881
JP8127	JPN	GI-18	AY296744	1–1635 (S1)
53XJ-99II	CN	GI-18	KC577391	1–1629 (S1)
LX4	CN	GI-19	AY189157	1–3495
Sczy3	CN	GI-19	JF732903	20365–23862
QXIBV	CN	GI-19	AF193423	1–1657 (S1)
Qu16	CN	GI-20	AF349620	1–1630 (S1)
Qu_mv	CN	GI-20	AF349621	1–1629 (S1)
Italy-02	ITA	GI-21	AJ457137	1–1620 (S1)
Spain/98/313	ES	GI-21	DQ064808	1–1614 (S1)
CK/CH/LSC/99I	CN	GI-22	DQ167147	1–1626 (S1)
HN08	CN	GI-22	GQ265940	1–1626 (S1)
GX-YL5	CN	GI-22	FJ907238	1–1621 (S1)
IBV/Ck/EG/CU/4/2014	EG	GI-23	KY805846	20360–23851
Variant 2	IL	GI-23	AF093796	1–1614 (S1)
V13	IND	GI-24	KF757447	1–1523 (S1)
IBV506	IND	GI-24	KF809796	1–1617 (S1)
GA/10216/2010	USA	GI-25	KM660636	1–1617 (S1)
GA/12274/2012	USA	GI-25	KP085595	1–1602 (S1)
NGA/BP61/2007	NG	GI-26	FN182268	1–1611 (S1)
NGA/N545/2006	NG	GI-26	FN182270	1–1611 (S1)
Georgia 08	USA	GI-27	GU301925	1–1630 (S1)
GA/12341/2012	USA	GI-27	KM660634	1–1647 (S1)
GX-NN-13	CN	GI-28	JX291989	1–1617 (S1)
SC021202	CN	GI-28	AY237817	1–1855 (S1)
γCOV/ck/China/I0118/14	CN	GI-29	KY407558	20371–23877
γCoV/ck/China/I0114/14	CN	GI-29	KY407556	20371–23877
D1466	NLD	GII-1	M21971	16–531 (S1)
V1397	NLD	GII-1	M21968	16–531 (S1)
N1/88	AU	GIII-1	U29450	78–1712 (S1)
V18/91	AU	GIII-1	U29521	78–1709 (S1)
DE/072/92	USA	GIV-1	AIU77298	1–1654 (S1)
AR/6386/97	USA	GIV-1	AF274436	1–1602 (S1)
N1/03	AU	GV-1	FJ235194	1–1627 (S1)
018	AU	GV-1	JX018208	1–1703 (S1）
TC07-2	CN	GVI-1	GQ265948	1–1638 (S1)
CO8089L	COL	GVI-1	AY604553	1–300 (S1)
GX-NN160421	CN	GVI-1	MW222189	20371–23883
GX-YL150727	CN	GVII-1	MK887061	1–1611 (S1)
CK/CH/2018/GZ04	CN	GVII-1	MN509336	1–1614 (S1)
CK/CH/LAH/08I	CN	GVII-2	GQ258302	1–1801 (S1)

^*a*^ Netherlands (NLD), the United States (USA), Republic of Indonesia (IND), China (CN), Australia (AU), New Zealand (NZL), Brazil (BRA), the United Kingdom (UK), Belgium (BEL), the Federal Republic of Nigeria (NG), Morocco (MA), the Republic of Colombia (COL), Israel (IL), Egypt (EG), Spain (ES).

### Determination of viral titer of the isolated strain

A total of 500 µL of IBV isolated strain allantoic fluid was inoculated into CEK cells in a 5 mL T25 cell culture flask. The cells were blind-passaged until the cytopathic effect (CPE) was observed. The time and degree of the lesions were recorded, and the cells and culture supernatant were harvested. The harvested samples were frozen and thawed three times, followed by centrifugation at 4,000 r/min for 10 min. The resulting supernatant was then inoculated into a 96-well cell culture plate containing a full monolayer of CEK cells to determine the TCID_50_ of the virus. The virus content was calculated using the Reed-Muench method. To investigate the growth characteristics of the IBV isolate in SPF chicken embryos, the allantoic fluid was diluted 10 times to 10^0^–10^−8^. Each embryo was inoculated with 0.1 mL of the diluted dose, and a group of five SPF chicken embryos was used for each dilution. The embryos were observed for 7 days, and the EID_50_ was determined based on the chicken embryo lesion situation. The experiment was repeated three times, and the average value was calculated. Infection was determined by the presence of chicken embryo death, dehydration, contraction, weak development, kidney enlargement, or urate deposition. The virus titer was calculated using the Reed and Muench method.

### Pathogenicity experiment

A total of 60 SPF chickens were randomly divided into three groups: IBV/CN/GD20230501 group, IBV-M41 group, and blank control group. The infection groups were infected with 10^5^ EID_50m_ through the nasal-ocular route, while the control group was given sterile PBS in the same manner. Daily observations and recordings were made for clinical signs. At 7, 12, 17, and 22 dpi post-infection, five chickens from each infection group were euthanized and dissected. The trachea collected at 7 dpi was fixed with 4% formaldehyde for histopathological analysis. Tracheal, nasal sinus swabs, lung tissue, kidney tissue, and cloacal swabs were collected for viral load detection through real-time qPCR. Blood samples were collected at 7, 12, 17, and 22 dpi from both the infected and control chickens. The specific antibodies against IBV in the serum were detected using an ELISA kit (Biosino, China) following the manufacturer’s instructions. The OD was measured at 450 nm using an automatic enzyme marker (Thermo Multiskan SkyHigh, USA).

### Real-time PCR quantitative analysis

Viral RNA was extracted from tracheal, lung, and kidney samples using the MiniBEST RNA/DNA Extraction Kit version 5.0 (TaKaRa, Dalian, China). All the clinical tissue homogenates (20%, wt/vol) were pooled and resuspended in phosphate-buffered saline (pH 7.2), vortexed, and then centrifuged at 12,000 × *g* at 4°C for 5 min. The final concentration was calculated as the copy number per gram of tissue sample. Primers were designed based on a conserved region of the N gene. The forward primer sequence was 5′-TTGAAGGTAGYGGYGTTCCTGA-3′, the reverse primer sequence was 5′-CAGMAACCCACACTATACCATC-3′, and the specific probe sequence was FAM-ACTGGAACAGGACCAGCCGCTGACCT-BHQ1. A standard plasmid, constructed using specific primers, was used as a positive reference. A standard curve was established to determine the conversion between the CT values and copy numbers of the subsequent detection results. The reaction conditions were as follows: 10 µL of 2× One Step RT-PCR Buffer III, 0.4 µL of Ex Taq HS (5 U/µL), 0.4 µL of PrimeScript RT Enzyme Mix II (RNA/DNA), 0.4 µL of each primer (20 pmol/µL) and probe, 2 µL RNA, and distilled water to a total volume of 20 µL. The amplification cycles consisted of incubation at 42°C for 5 min, followed by denaturation at 95°C for 10 s. This was followed by 40 cycles of denaturation at 95°C for 5 s and annealing/extension at 59°C for 34 s. Fluorescent signals were measured at the end of each cycle. Each reaction was performed in triplicate, and the results were reported as the average ± standard deviation (SD).

### Metabolomics analysis

At 7 days post-infection, three randomly selected samples from each group were collected from the wing, and the serum was separated for non-targeted metabolomics detection analysis. The analysis was conducted using a Thermo Vanquish Ultra-High Performance Liquid System (Thermo Fisher Scientific, USA) with an ACQUITY UPLC HSS T3 chromatographic column (2.1 × 100 mm, 1.8 µm) (Waters, Milford, MA, USA). The flow rate was set at 0.3 mL/min, the column temperature was maintained at 40°C, and the injection volume was 2 µL. In positive ion mode, the mobile phase was 0.1% formic acid acetonitrile (B2) and 0.1% formic acid water (A2), and the gradient elution program was as follows: 0–1 min, 8% B2; 1–8 min, 8%–98% B2; 8–10 min, 98% B2; 10–10.1 min, 98%–8% B2; and 10.1–12 min, 8% B2. In negative ion mode, the mobile phase was acetonitrile (B3) and 5 mM ammonium formate water (A3), and the gradient elution program was as follows: 0–1 min, 8% B3; 1–8 min, 8%–98% B3; 8–10 min, 98% B3; 10–10.1 min, 98%–8% B3; and 10.1–12 min, 8% B3 (55). Detection was performed on a Thermo Orbitrap Exploris 120 mass spectrometer (Thermo Fisher Scientific, USA) equipped with an electrospray ion source. Data were collected in both positive and negative ion modes. The positive ion spray voltage was set at 3.50 kV, while the negative ion spray voltage was set at −2.50 kV. The sheath gas was maintained at 40 arb, and the auxiliary gas at 10 arb. The capillary temperature was set to 325°C. The first-level full scan was conducted at a resolution of 60,000, covering a range of *m/z* 100–1,000. Secondary fragmentation was carried out using HCD with a collision energy of 30%. The secondary resolution was set at 15,000, and fragmentation was performed on the four ions prior to signal collection. Dynamic exclusion was employed to remove unnecessary MS/MS information (56).

The original mass spectrometry offline files were converted to the mzXML file format using the MSConvert tool in the Proteowizard software package (v3.0.8789) (57). Peak detection, peak filtering, and peak alignment processing were performed using the R XCMS (v3.12.0) software package to obtain a metabolite quantification list (58). The parameter settings for this process were bw = 2, ppm = 15, peakwidth = c (5, 30), mzwid = 0.015, mzdiff = 0.01, and method = “centWave.” Subsequently, data correction was achieved by normalizing through the total peak area to eliminate systematic errors. Substance identification was conducted by searching and comparing with spectral databases such as HMDB (59), massbank (60), LipidMaps (61), mzcloud (62), KEGG (63), and the self-built metabolite standard substance database of Nome Metabolism. The parameter setting for this search was ppm < 30 ppm. The MetaboAnalyst software package was utilized for functional pathway enrichment and topological analysis of the screened differential metabolites (31). MetaboAnalyst (www.metaboanalyst.ca) was utilized for conducting KEGG pathway enrichment analysis on the differential metabolite lists. The enrichment method is grounded on the hypergeometric distribution test, while the topological analysis employs the degree centrality method. The objective of topological analysis is to assess the significance of a gene or metabolite in a biological reaction by considering its placement within the pathway.

### Statistical analysis

Statistical analysis was performed using GraphPad Prism 9 (GraphPad Software Inc., San Diego, CA, USA) to analyze viral titers. Descriptive statistics such as mean and SD were used. Student’s *t*-test was employed for normally distributed variables. Comparisons of viral genome copy numbers in chicken tissues infected with different viruses at each time point were made, considering *P* < 0.05 as statistically significant differences. Two different multivariate statistical analysis models, unsupervised and supervised, were utilized to distinguish the groups (PCA, PLS-DA, and OPLS-DA) through the R ropls (v1.22.0) package (64). The statistical significance of *P* value was determined by conducting a statistical test between the groups. Finally, biomarker metabolites were identified by combining *P* value, VIP (OPLS-DA variable projection importance), and FC (multiple of differences between groups). The data were analyzed on the BioDeep Platform (http://www.biodeep.cn).

## Data Availability

The sequences of IBV/CN/GD20230501 have been deposited in GenBank under the accession number OR778292.

## SUPPLEMENTAL MATERIAL

Table S1 spectrum.03990-23-s0001.xlsx Results of half lethality of IBV/CN/GD20230501 strain on CEK cells.

Table S2 spectrum.03990-23-s0002.xlsx EID 50 determination in the IBV/CN/GD20230501 strain.

Table S3 spectrum.03990-23-s0003.xlsx 7,648 differential metabolites were identified overall.

Table S4 spectrum.03990-23-s0004.xlsx The identification of 291 metabolites.

Table S5 spectrum.03990-23-s0005.xlsx Results of the differential metabolite pathway analysis.

ASM does not own the copyrights to Supplemental Material that may be linked to, or accessed through, an article. The authors have granted ASM a non-exclusive, world-wide license to publish the Supplemental Material files. Please contact the corresponding author directly for reuse.
